# Pharyngeal carriage rates of *Neisseria meningitidis* in health care professionals at a tertiary university pediatric hospital

**DOI:** 10.1007/s10096-020-03894-9

**Published:** 2020-04-24

**Authors:** Lisa-Maria Steurer, Mathias Hetzmannseder, Birgit Willinger, Peter Starzengruber, Claudia Mikula, Andrea Kormann-Klement, Michael Weber, Angelika Berger, Agnes Grill

**Affiliations:** 1 Department of Pediatrics and Adolescent Medicine, Division of Neonatology, Pediatric Intensive Care Medicine and Neuropediatrics, Medical University of Vienna, Vienna, Austria; 2 Department of Laboratory Medicine, Division of Clinical Microbiology, Medical University of Vienna, Vienna, Austria; 3 Institute for Medical Microbiology and Hygiene, Austrian Agency for Health and Food Safety, Graz, Austria; 4 Section for Medical Statistics, CeMSIIS, Medical University of Vienna, Vienna, Austria

**Keywords:** *Neisseria meningitidis*, Pharyngeal carriage rates, Pediatric health care professionals, Meningococcal carriage, Pediatric hospital

## Abstract

Pharyngeal carriage is the reservoir for *Neisseria meningitidis* in the population and the first step in disease transmission. Especially in young infants and adolescents, *N. meningitidis* can cause serious invasive infection with high fatality rates and high rates of long-term sequelae among survivors. The aim of this study was to determine *N. meningitidis* colonization rates in asymptomatic health care professionals at a tertiary university pediatric hospital and to identify risk factors for carriage. This cross-sectional meningococcal carriage survey was conducted between April and October 2018 at the Medical University of Vienna. Individuals working as nurses, pediatricians, or medical students were enrolled. Oropharyngeal swabs were directly plated onto selective agar plates and conventional culture was used for bacterial identification. Meningococcal isolates were further characterized using whole-genome sequencing. A total of 437 oropharyngeal specimens were collected. Overall, meningococcal carriage prevalence was 1.14% (5/437), with 0.7% (3/437) for capsular genotype B, and 0.5% (2/437) for capsular genotype W. Mean age of carriers was significantly lower than of non-carriers (24.2 vs. 35.8; *p* = 0.004). The highest carriage rate of 4.4% (4/91) was found in the age group 18–25. Carriage was negatively associated with age and timespan working in pediatrics. This is the first study evaluating the prevalence of *Neisseria meningitidis* carriage in health care professionals working in Pediatrics and Adolescent Medicine. Carriage was in general lower than expected for all age groups, implicating a low risk of meningococcal transmission via this population.

## Introduction

*Neisseria meningitidis* is a diplococcal, aerobic, Gram-negative, and obligate human bacterium categorized into 12 different serotypes according to capsular polysaccharide structure. The epidemiologically most relevant serogroups are A, B, C, W, and Y accounting for > 90% of invasive meningococcal disease (IMD) cases worldwide [1].

It can colonize the human nasopharynx as part of its complex microbiota without affecting the host. Carriage is a highly variable phenomenon and differs with age, region, and setting [2]. Lowest carriage rates were found in newborns and infants, then it increases throughout childhood, peaking in adolescence and young adult age, before gradually declining again in older age [3]. In closed or semi-closed populations such as military recruits and university students living in campuses, or in disease outbreak settings, it can reach up to 25% or even more [4].

As the human nasopharynx is the unique biological niche for *N. meningitidis*, the carrier state is regarded as the reservoir for meningococcal transmission being passed from one person to the other via respiratory droplets, direct contacts with IMD patients, or more often asymptomatic carriers.

Acquisition usually results in asymptomatic carriage, but in rare cases, the bacteria can escape the mucosal barrier and invade the bloodstream leading to lifethreatening conditions such as meningitis and sepsis with letality rates of 10–15% and resulting in severe long-term sequelae in up to 20% of survivors [5]. Hence, it remains an important public health issue globally, despite a generally low overall IMD incidence of 0.6 annual cases per 100,000 inhabitants across Europe [6].

Highest disease incidences have consistently been found in the pediatric patient cohort, i.e., in infants in the first year of life when maternal antibodies progressively fade out, in 1–4-year-old children, and in adolescents [7]. Infants are regarded most susceptible to severe IMD due to their immature immune system, whereas adolescents are at risk because of high carriage and transmission rates.

An elevated risk for meningococcal infection and disease has also been described for health care workers exposed to patients with meningococcal disease. The excess risk for physicians, nurses, or paramedics with intensive close contact during airway management or mouth-to-mouth resuscitation of infected cases is estimated 25 times that in the general population [8]. The majority of occupationally acquired meningococcal disease, however, occurred in the setting of improper precaution, i.e., unprotected exposure to infected patients without use of post-exposure chemoprophylaxis [9, 10].

Carriage studies are important to understand the epidemiology and pathogenesis of meningococcal disease and for establishing vaccination strategies, since vaccination of population cohorts with highest carriage rates has been proven most effective to confer herd protection [11].

This is especially important in medical staff working in pediatrics treating the patient cohort the most susceptible and vulnerable to severe meningococcal disease. Currently, there is no surveillance of asymptomatic carriers in this population.

In this cross-sectional carriage study, we aimed to assess meningococcal carriage rates in a health care personnel cohort working in pediatrics and adolescent medicine where there is permanent contact to patient cohorts with the highest risk for meningococcal carriage and disease.

## Materials and methods

### Study design and participants

This study was conducted as a cross-sectional carriage survey evaluating *Neisseria meningitidis* colonization in asymptomatic health care professionals at the department of pediatrics and adolescent medicine of the Medical University of Vienna during a 6-month study period from 20 April–30 October 2018. All medical staff members working as nurses or doctors on 9 different pediatric wards, as well as medical students passing an internship for at least 4 weeks, were recruited for participation on voluntary basis. Exclusion criteria were antibiotic therapy in the preceding 4 weeks and working less than 4 weeks in pediatrics. Although not assessed formally, approximately 10–20% of eligible individuals refused participation.

### Specimen and data collection

Prior to sample collection, each volunteer signed a written informed consent form and was administered a de-identified self-administered questionnaire consisting of socio-demographic data, e.g., sex, age, profession, time span working in pediatrics, main work setting (neonatology, pediatric intensive care unit, outpatient department, general pediatric unit), and information on potential risk factors associated with meningococcal carriage, such as active smoking (defined as smoking at least one cigarette daily), recent symptoms of upper respiratory tract infection in the last 2 weeks, number of household members, number and age of children/adolescents living in the same household, and recent or past contacts with a patient with IMD [12–14]. Furthermore, the questionnaire collected the meningococcal vaccination status of participants.

Oropharyngeal samples were collected by trained personnel using a sterile flocked nylon swab (FLOQSwabs/ESwab™, Copan Diagnostics Inc., Brescia, Italy). One single swab was taken from the posterior wall of the oropharynx behind the uvula via the mouth of each subject using a standardized technique [15] and was immediately plated onto a selective agar medium (Chocolate agar + PolyViteX VCAT3, described by Thayer & Martin, bioMérieuxSA, Marcy l’Étoile, France) and on Columbia Blood Agar.

### Laboratory procedures and bacterial identification

The collected plates were processed at the microbiological laboratory of the Medical University of Vienna, where the media were incubated within 3 h at 37 °C in 5% CO_2_ and examined at 18–24 h and at 48 h for the growth of *N. meningitidis.* All colonies suspected for being *N. meningitidis* underwent gram-staining and additional identification with matrix-assisted laser desorption/ionization time-of-flight mass spectrometry analysis (MALDI-Biotyper, Bruker, Germany) according to the manufacturer’s instructions.

All confirmed isolates were sent to the Austrian National Reference Center for Meningococcal Disease in Graz (AGES), where identification of serogroups was performed using slide agglutination and whole-genome sequencing (WGS). The WGS was performed on purified genomic DNA by parallel high-throughput next-generation sequencing (NGS) technologies on Illumina MiSeq platform (Illumina Inc., San Diego, CA, USA) according to the manufacturer’s protocol.

Further analysis concerning sequence types, clonal complex, and outer membrane (PorA and FetA) variants and alleles of the meningococcal isolates was performed using the Neisseria species MLST database (https://pubmlst.org/neisseria) of the University of Oxford.

### Statistical analysis

All computations were performed using IBM SPSS Statistics for Windows version 23.0 (IBM, Armonk, NY).

Nominal data are presented using percentages as well as absolute frequencies. Metric data are presented using mean ± standard deviation. For the continous variable “age,” also median, interquartile range (IQR), and total range were determined; additionally, age was recoded into four age groups (18–25, 26–35, 36–45, > 45). Differences between the groups given categorical dependent variables were compared using two-sided Fisher’s exact test or chi2 test as appropriate [16]. In addition, crosstabs and Fisher exact tests were used to assess the correlation between risk factors and carriage rates. *p* values equal or below 5% were considered statistically significant.

#### Ethics statement

The study protocol, assent form, and the questionnaire were approved by the ethics committee at the Medical University of Vienna (2018-01-21, reg.-no. 2229/2017). Each participant signed written informed consent. Additional approval was obtained from the data protection committee and the employee organization of the Medical University Vienna.

## Results

### Participant characteristics and carriage prevalence

A total of 484 oropharyngeal samples were collected, of which 47 subjects had to be excluded according to our study protocol. Of them, 38 were working in pediatrics for less than 4 weeks, eight had antibiotic treatment in the preceding 4 weeks, and one had to be excluded due to a missing questionnaire and assent form.

The remaining 437 participants were included for further analysis and comprised 307 nurses, 110 doctors, and 20 medical students; 85.6% (374/437) were females and 14.4% (63/437) were males.

The mean age of our study population was 35.6 years (SD 10.7, median 33, IQR 17, total range 18–64 years); 34.9 years (SD 10.6) for nurses, 39.2 years (SD 10.3) for doctors, and 26.9 years (SD 3.5) for medical students.

The majority were non-smokers (85.4%, 373/437).

According to the questionnaire, 22.4% (98/437) had experienced symptoms of respiratory tract infection within 2 weeks prior to swabbing. Eight percent (35/437) reported contact to a patient with invasive meningococcal disease within 1 year prior to sampling.

Full demographic characteristics of the participants are shown in Table 1.

**Table 1:** Participant characteristics: demographic characteristics of total sample (*n* = 437) and *N. meningitidis* carriers (*n* = 5)

Category		Overall participants *n* (%)	Carriers *n* (%)
Total number		437 (100%)	5/437 (1.14%)
Profession	Nurses	307 (70.3%)	4/307 (1.3%)
	Doctors	110 (25.2%)	0/110 (0%)
	Medical Students	20 (4.5%)	1/20 (5%)
Age group (years)	18–25	91 (20.8%)	4/91 (4.4%)
	26–35	169 (38.7%)	1/169 (0.6%)
	36–45	89 (20.4%)	0
	> 45	88 (20.1)	0
Mean age (years)	Total	35.6 (10.7 SD), median 33	24.2 (4.6 SD), median 23
	Nurses	34.9 (10.6 SD) median 32	–
	Doctors	39.2 (10.3 SD) median 36	–
	Medical Students	26.9 (3.5 SD) median 27	–
Timespan working in pediatrics (years)	< 1	86 (19.7%)	4/86 (4.7%)
	1–5	127 (29.1%)	1/127 (0.8%)
	6–10	60 (13.7%)	1/60 (1.7%)
	> 10	164 (37.5%)	0
Gender	Female	374 (85.6%)	4/374 (1.1%)
	Male	63 (14.4%)	1/63 (1.6%)
Active Smokers		64 (14.6%)	1/64 (1.6%)
Resp tract infections (< 2 weeks)		98 (22.4%)	0
Mean household size (persons)		2.6 (1.2 SD), median 2.0 (1–8)	2.4 (1.1 SD), median 2.0 (1–4)
Recent IMD contact (< 1 year)		39 (8.9%)	0
Vaccination against *N. meningitidis* (any serogroup)	Total sample	117/411 (28.5%)	0
	Nurses	50/286 (17.5%)	0
	Doctors	57/107 (53.3%)	0
	Medical Students	10/18 (55.6%)	0
Vaccination against serogroup ACWY	Total sample	66/415 (15.9%)	0
	Nurses	21/289 (7.3%)	0
	Doctors	39/108 (36.1%)	0
	Medical Students	6/18 (33.3%)	0
Vaccination against serogroup B	Total sample	50/415 (12.0%)	0
	Nurses	9/289 (3.1%)	0
	Doctors	34/108 (31.4%)	0
	Medical Students	7/18 (38%)	0

Data on meningococcal vaccination status were available for 94% (411/437) of our study population and showed an overall vaccination rate of 28.5% (117/411). The meningococcal vaccination rate among nurses was 17.5% (50/286), among doctors 53.3% (57/105), and 55.6% (10/18) among medical students. Characteristics of participants’ meningococcal vaccination history are shown in Table 1.

Among the 437 pharyngeal samples, five *N. meningitidis* isolates were recovered, rendering an overall carriage rate of 1.14%; 0% in doctors (0/110), 1.3% in nurses (4/307), and 5% in medical students (1/20). Carriage prevalence was highest among the age group 18–25 years (4.4%, 4/91). None of the carriers was older than 35 years and none of them had any vaccination against meningococci.

Serogroup identification showed 3 serogroup B and 2 serogroup W isolates, accordingly 0.69% and 0.46% of the study population. We did not find other serogroups or unencapsulated meningococci.

Genotypic characteristics of recovered *N. meningitidis* isolates are shown in Table 2.

**Table 2:** Molecular characterization of the five recovered *N. meningitidis* isolates: genogroup, sequence types, clonal complex, and outer membrane variants (PorA and FetA)

Serogroup	Capsule group	ST	Clonal complex	PorA VR1	PorA VR2	FetA VR
B	B	213	ST-213 complex	22	14	F5–5
W	W	11	ST-11 complex	5	2	F1–1
W	W	10,076	ST-35 complex	22–1	14–40	F3–9
B	B	1572		18	25	F1–7
B	B	9661	ST-213 complex	22	14	F5–5

### Association with N. meningitidis carriage

*N. meningitidis* carriers were significantly younger than non-carriers (24.2 vs. 35.8; *p* = 0.004).

Univariate analysis revealed that age and the timespan working in pediatrics were significantly inversely associated to meningococcal carriage. In contrast, there was no statistically significant association between carriage and recent contact to an IMD patient, respiratory tract infection, smoking, and vaccination against any meningococcal serogroup. No significant differences in carriage were found between different professions and main work settings. In addition, no differences in detection rate could be found according to the month of swab collection. The number of participants with carriage (*n* = 5) was too small to perform a multivariable model or to assess an interaction effect between variables.

## Discussion

To our knowledge, this is the first study evaluating *N. meningitidis* carriage rates in asymptomatic health personnel working in pediatrics and adolescent medicine. In addition, sparse data are available for health care workers in general.

The main finding was a low overall meningococcal carriage prevalence of 1.14%. This low prevalence is possibly related to the characteristics of our study population, showing a low-risk profile according to previously reported risk factors associated with meningococcal carriage: advanced age of study participants, low number of smokers, 85% of the study population being female, and high socioeconomic status [1]. Active smoking is a well-recognized risk factor for meningococcal carriage, since it causes structural changes in the respiratory tract mucosa and impairs immune response [17, 18]. Male gender is associated with significantly higher carriage rates in most carriage studies [19].

However, we believe that the most important aspect in our cohort is the relatively high mean age of participants being 35.6 years compared to most meningococcal carriage studies. Even the mean age of students was 26.9 years. The carriage prevalence found among individuals aged 18–25 years, who are more often the target group in meningococcal carriage studies, was 4.4% (4/91) in our cohort and hence significantly higher than in older age groups. This finding is well in line with the literature, as highest carriage rates have consistently been found in this age group [3]. This effect seems to be most likely due to a convergence of risk factors and social habits that facilitate close interpersonal contacts observed in young adults than age per se [20, 21]. Studies comparing carriage rates of young adults at entry of university or military service to those later in the academic year or at the end of military service observed much lower carriage prevalence at the beginning of university or military service [22, 23]. For example, a carriage survey of military recruits aged 18–24 years in Finland showed a low overall meningococcal carriage rate of 2.2% (20/892) among those beginning military service, compared to 18.5% (151/818) at the end of their military service [24].

Most meningococcal carriage studies have been undertaken in at-risk populations, i.e., teenagers, university students, military recruits, disease outbreak settings, and Hajj pilgrims [2, 3, 19]. *N. meningitidis* carriage in older individuals, i.e., beyond the teenage years and young adult age, especially in low-risk settings, is poorly understood, primarily due to a dearth of carriage data available and hence meningococcal carriage in the total population might have been overestimated.

A recent carriage survey among individuals aged 65 years and older in Germany found a remarkably low prevalence of colonization with *N. meningitidis* of 0.3% (2/677; 95% CI 0–1.1%) [25]. These data suggest that carriage rates might dramatically drop in older age groups to a much bigger extent than previously thought. A cross-sectional study in Tuscany, Italy, found a carriage prevalence of 4.8% in individuals aged 11–45 years presenting at immunization clinics. Among the age group 31–45 years, the carriage prevalence was 2.4% (20/828), while it was 9% (37/434) in the age group 20–30 years [26]. A study conducted in 2014 in Hajj pilgrims with a mean age of 50 years found 1.3% meningococcal carriers in individuals originating from low endemic countries, while 8.9% of pilgrims from high endemic regions (sub-Saharan meningitis belt) were tested positive for *N. meningitidis* [27].

Other recent studies also described lower meningococcal carriage prevalence than anticipated even in high-risk settings. In a carriage survey in Greece in 2014/2015 including 1420 individuals aged 18–26 years, Tryfinopoulou et al. observed significantly lower carriage prevalences compared to their previous studies 20 years ago among military recruits (15% vs. 25%, *p* < 0.05) and university students (10.4% vs 18%, *p*=0.002) [28].

Carriage rates in university students aged 17–25 years in South Australia were 6.3% and hence lower than anticipated [29]. Authors conclude that possible factors for lower meningococcal carriage rates might be a reduction to smoking habits and meningococcal vaccination.

Significant impact on carriage rates has been reported for most meningococcal conjugate vaccines [30–32]. In Austria, meningococcal vaccination is recommended for personnel working in pediatric facilities because of an elevated risk of exposure to IMD cases [33]. In our subcohort of doctors, there was a high meningococcal vaccine coverage of more than 50%; in this subcohort, we did not find any *N. meningitidis* carrier, while none of the detected carriers in our study population had any vaccination against meningococci. However, this effect was not statistically significant.

Meningococcal carriage prevalence among medical staff members in general has not largely been investigated, and information concerning this cohort is limited to isolated reports. Coch Goia et al. tested 200 staff members in a university hospital in Brazil. Carriage prevalence in the hospital employee cohort (mean age 41.9) was 3% (3/100), while it was 15% (15/100) in students (mean age 23.6). These findings again suggest age as the predominant factor for carriage [34].

The estimated relative risk concerning work-related IMD is increased for health care providers exposed to respiratory droplets of affected patients compared to the general population [8]. The majority of published meningococcal infections in health care workers, however, occurred in the setting of improper precautionary measures. Using appropriate personal protective equipment of any kind and post-exposure chemoprophylaxis, the risk for occupational meningococcal transmission resulting in either secondary meningococcal disease or carriage is estimated to be low [9, 10].

Despite all aforementioned aspects, our detected carriage rates are still less than anticipated even for the youngest age group investigated compared to most of the pre-existing literature. Key limitation in interpreting the presented results is that a comparable cohort of pediatric clinic employees has not yet been investigated.

Factors that might hypothetically be special in a pediatric health personnel cohort include frequent contact to IMD cases with subsequent post-exposure prophylaxis that also effectively eradicates colonization with any meningococci [35]. In addition, unknown factors of social behavior patterns in this cohort might contribute.

This is not only the first carriage study in a pediatric health personnel setting but also the first meningococcal carriage study in Austria. The incidence of IMD in Austria is among the lowest in Europe. There has been a gradual decline over the past 10 years; in 2018, the IMD incidence was 0.34 per 100,000 inhabitants [36]. This low national disease incidence might be an additional explanation for the low carriage prevalence found, as it is assumed to find higher carriage rates in high endemic countries [27].

Sample collection from April to October without collecting swabs during winter should not have had an impact on carriage rate, especially considering that the mean duration of meningococcal carriage is estimated to range from 3.4 to 11.7 months [37, 38]. Unlike meningococcal disease, carriage does not seem to be relevantly influenced by seasonality according to the literature [2, 13].

In terms of serogroup distribution, we found serogroup B and serogroup W in carriers. Serogroup B is usually the dominant capsular group in carriage studies across Europe and in most countries worldwide, while serogroup W was historically less frequently detected in carriage studies since those strains seem to have a shorter duration of carriage [2, 17]. However, during the last years, an increase in MenW IMD was observed in most European countries, following emerging MenW IMD cases in South America since 2004 and hence MenW is detected more frequently among carriers [39]. In a recent carriage study in Turkey, MenW accounted for 66.6% of all recovered meningococcal isolates [21].

Interestingly, we did not observe any non-groupable meningococci, although non-encapsulated *N. meningitidis* are frequently found among carriers according to the literature [26, 40–42].

This cross-sectional carriage survey has several limitations. First, using one single swab might have underestimated carriage rates, since the sensitivity of swabbing is estimated to be 60–83% [43]. Out of respect for our volunteers and to keep attrition rates low, we decided to take only one pharyngeal swab. In addition, our study was substantially limited by the unexpected small number of carriers, so that our analysis did not have enough power to assess significance of potential associations or risk factors for carriage. Further limitations were the lack of a control group and of longitudinal data.

Methodically, we decided for direct plating of swabs followed by conventional culture methods for bacterial identification, although recently reported methods using PCR after THB (Todd-Hewitt broth) culture might have had a higher yield [44]. However, this is not standard yet. Conventional culture has been used in the majority of carriage studies, which allows better comparison of results but might bear the risk of underestimating the true level of carriage [2, 3]. With the possibility of collecting swabs in a university hospital setting with immediate availability of microbiological processing of collected plates, we were able to avoid altering results due to transport delays.

Despite aforementioned limitations, our study provides important new aspects concerning the epidemiology of *N. meningitidis* in pediatric hospitals. Our results show that the prevalence of pharyngeal meningococcal colonization is low among health care personnel working in pediatrics and adolescent medicine, and thus, the risk of meningococcal transmission is low.
